# Disease Dynamics in a Specialized Parasite of Ant Societies

**DOI:** 10.1371/journal.pone.0036352

**Published:** 2012-05-02

**Authors:** Sandra B. Andersen, Matthew Ferrari, Harry C. Evans, Simon L. Elliot, Jacobus J. Boomsma, David P. Hughes

**Affiliations:** 1 Centre for Social Evolution, Department of Biology, University of Copenhagen, Copenhagen, Denmark; 2 Center for Infectious Disease Dynamics, Penn State University, University Park, Pennsylvania, United States of America; 3 CAB International, E-UK, Egham, Surrey, United Kingdom; 4 Department of Entomology, Federal University of Viçosa, Viçosa, Brazil; 5 Department of Entomology and Department of Biology, Penn State University, University Park, Pennsylvania, United States of America; University of Sheffield, United Kingdom

## Abstract

Coevolution between ant colonies and their rare specialized parasites are intriguing, because lethal infections of workers may correspond to tolerable chronic diseases of colonies, but the parasite adaptations that allow stable coexistence with ants are virtually unknown. We explore the trade-offs experienced by *Ophiocordyceps* parasites manipulating ants into dying in nearby graveyards. We used field data from Brazil and Thailand to parameterize and fit a model for the growth rate of graveyards. We show that parasite pressure is much lower than the abundance of ant cadavers suggests and that hyperparasites often castrate *Ophiocordyceps*. However, once fruiting bodies become sexually mature they appear robust. Such parasite life-history traits are consistent with iteroparity– a reproductive strategy rarely considered in fungi. We discuss how tropical habitats with high biodiversity of hyperparasites and high spore mortality has likely been crucial for the evolution and maintenance of iteroparity in parasites with low dispersal potential.

## Introduction

Specialized parasites that interact with a single or narrow spectrum of hosts tend to have fascinating life-histories, because virulence and defence traits are likely to have been shaped by co-evolutionary arms races [1], [2]. This is particularly true for parasites that have evolved ways to manipulate host behaviour, so that dying hosts express extended phenotypes that serve parasite reproductive success [3], [4]. The fungal hypocrealean genus *Ophiocordyceps* (formerly *Cordyceps*) is well known for attacking specific hosts from diverse insect orders [5]. Several lineages have evolved species that attack ants [6] leading to behavioural extended phenotypes, i.e. host manipulation, to make infected ants leave their nests to die and disperse spores in ways that serve parasite fitness [7].

Ant colonies are peculiar hosts for parasites. Following a high mortality rate at the founding stage, mature colonies of some species such as leaf-cutting ants, harvester ants and some *Formica* and *Camponotus* species [8] are extraordinarily long-lived, which is suggested to be correlated with low extrinsic mortality once colonies are mature [9]. The high density of continuously interacting individuals within colonies implies that infection risks are high [10], [11], but also that selection for efficient prophylactic defences has been strong [12], [13], [14]. Recent reviews [15], [16], [17] have emphasized that behavioural forms of social immunity are normally very efficient, so that ant parasites pose a limited threat for escalating epidemics within colonies. Thus, even though individual ants may die from infection, theory predicts that disease-induced mortality of mature ant colonies should be low, albeit evidence is only available for some species [17].

Horizontal disease transmission to new colonies requires the introduction of parasite propagules to uninfected nests. Long-lived propagules may be encountered in the nesting material while other types can only reach the modest percentage of workers that are out foraging (typically between 10–25% of the workers) [18], [19], [20], even though intra-colonial transmission by worker-worker interactions could occur. Environmental transmission may not be very efficient as territoriality often limits overlap between infected and susceptible colonies [14]. However, if chronically infected ant colonies tend to be long-lived, a combination of frequent intracolony (nestmate to nestmate) infection and rare horizontal transmission across colonies may secure stable host-parasite interactions in ants [12], [21], [22], [23].

Here we set out to examine the dynamics of the interaction between ant hosts and *Ophiocordyceps* parasites, which all available evidence show are highly specialized [24], in tropical forests. The fungus manipulates workers to leave their nest to die close to their host colony in high-density graveyards that may persist on the same location for years, offering the advantage that mortality rates due to chronic parasitism can be estimated [25], [26], [27], [28]. Apart from the intriguing extended phenotype adaptations that allow the fungus to control ant behaviour, *Ophiocordyceps* fungi that exploit ants are also unusual in that the major growth phase and all parasite reproduction occurs long after host death. The fruiting body of the parasite has a latent period of at least two weeks before it reproduces (release mature spores) for the first time, and the fungus secures the ant cadaver so efficiently that it can continue with successive bouts of reproduction without succumbing to decay [7]. This implies that *Ophiocordyceps* have life-history traits reminiscent of perennial plants, including traits such as age at first reproduction and allocation to current *versus* future reproduction that have been shaped by selection and are likely to be linked to rates of ageing and investment in somatic repair [29].

In a classic paper, Charnov and Schaffer [30] showed that iteroparous life cycles with continuing investment in somatic tissue can only evolve when juvenile mortality is high relative to adult mortality. To our knowledge, the applicability of this logic has never been explicitly tested in fungi (where iteroparous fruiting bodies are rare with the exception of some saprotrophic fungi [31]), but available natural history data suggest that *Ophiocordyceps* may well have the appropriate combination of traits for this conceptual framework to apply. *Ophiocordyceps unilateralis s.l.* species (as studied here) all have aerially projected spores that are thin-walled and hyaline (clear) and thereby susceptible to desiccation and UV damage [24]. While they may have the ability to acquire pigmentation (H.C. Evans, unpublished) they appear not to be built for persistence, suggesting that spores do not remain viable for extended periods in the environment following ejection [24]. Somatic investment to secure continued growth of the fruiting body is substantial in the only species studied in detail so far, *O. unilateralis s.l*. [7]. However, a variety of fungal hyperparasites colonize the developing stalks and fruiting bodies and potentially cause effective castration [32]. The Charnov and Schaffer model would not be supported if, in spite of investments in somatic maintenance, only spores produced shortly after sexual maturity of fruiting bodies would pass on genes to future parasite generations. It is essential, therefore, to know the relative rates at which fruiting bodies become reproductively dysfunctional in their early phases of development.

Applying iteroparity life-history theory to a specialized host-parasite interaction such as *Ophiocordyceps* has interesting additional complications, as intracolony transmission success may, paradoxically, limit population-wide reproductive success of parasites no matter whether spores are produced directly after sexual maturity of a fruiting body or long after that. Recurrent intracolony transmission requires a minimum number of dead ants per unit of time and a particular rate of infectivity to maintain a local population, whereas host colonies need to be large enough to sustain the ensuing level of worker mortality without going extinct. When mortality happens in ‘graveyards’, this would require that the graveyards have a growth rate above one (i.e. net inflow rate of dead infected ants) or, in case of long-term equilibrium with the population of host ants, a growth rate equal to one.

In the present study, we used data from previous studies on *O. unilateralis* in Thailand [7], [27] and a new data set from *O. camponoti-rufipedis* [ = *O. unilateralis s.l.*, 24] from Brazil to parameterize a developmental-stage-structured model describing the interaction dynamics between *Ophiocordyceps* and its host ants. By measuring the distribution of parasite life stages and the occurrence of hyperparasitism within ant graveyards we estimated the actual parasite pressure on the ants. We show that most parasite fruiting bodies are incapable of transmitting infectious propagules because of hyperparasitism, but that iteroparous reproduction appears essential for maintaining marginally positive growth rates in ant graveyards. Our results suggest that slow development of fruiting bodies and iteroparous reproduction are likely to be adaptations that achieve long-term persistence with host-ant colonies. This is because infection success of spores is likely to be low as new host ants are difficult to target in both time and space, so that prolonged survival of fruiting bodies increases parasite reproductive success in spite of relatively high costs of hyperparasitism.

## Materials and Methods

### Fieldwork

The common ant *Camponotus rufipes* is host to the specialized parasite *Ophiocordyceps camponoti-rufipedis* in the Atlantic rainforests of Brazil [24]. The ants form large, long-lived colonies headed by a single queen (monogyny) that have been observed to survive at the same site for more than 10 years (R.F. de Souza & S. Robeiro, personal communication). The ants construct nests of leaves, twigs and soil, typically at the base of trees and often connected to smaller satellite nests, and forage at night along temporally stable trails. The overall distribution of *O. camponoti-rufipedis* has been studied since 2006 and provided the stimulus for the present focal study, undertaken in February 2011 in Mata do Paraíso, a 400 ha Atlantic rainforest nature reserve in Minas Gerais, Brazil. The forest harbours a high density and diversity of *Ophiocordyceps* that infect ants, of which *O. camponoti-rufipedis* is one of the most common [24].

We identified ants infected with *O. camponoti-rufipedis* by searching the underside of leaves along a *ca.* 460 m stretch of forest path and found five graveyards [27] with a high density of dead *C. rufipes*, each of them situated around a single host ant colony. We marked areas covering approximately the entire graveyard (graveyard 1, 2, 3) or large parts of them (graveyard 4, 5), and tagged all dead infected ants - found typically on the underside of leaves and on twigs (n = 432) - with pink tape around the leaf stem. After death, *Ophiocordyceps* parasitized ants progress through several developmental stages. For each cadaver we therefore characterized the state of parasite development as being: 1. a freshly killed ant, 2. a cadaver with a parasite stroma (stalk-like structure that develops into a mature fruiting body), 3. a cadaver with a mature parasite sexual fruiting body (ascoma), 4. a cadaver at stage 2 or 3, but hyperparasitized by other fungi, or 5. a cadaver whose status could not be identified as it had been damaged by unknown causes and lacked obvious fungal growth (see below for more detailed category descriptions). The coordinates of each graveyard were obtained with a Garmin *etrex* GPS and mapped in GoogleEarth.

### Measuring fungal reproduction

To estimate the infectivity of mature parasite fruiting bodies, 15 dead ants with developed sexual reproductive bodies (ascomata) were collected with the leaves they were attached to and suspended on a wooden platform above a microscope slide in the forest close to, but outside, a graveyard of dead infected ants. Spore discharge takes place during the night and microscope slides were therefore checked on the three following mornings for deposition of the highly distinctive spore clouds, which are visible to the naked eye [24].

After this collection period, the 15 parasitized ants with mature fruiting bodies were brought to the lab, in addition to 16 newly collected dead ants with mature fruiting bodies. All 31 ants were individually attached with Vaseline to the lid of a Petri dish with a microscope slide or agar at the bottom and placed in a dew chamber with 100% relative humidity for 18 hours (from 18.00 to 12.00 the following day). The lab-generated microscope slides and agar plates were then checked for spore deposits every morning for 4–6 consecutive days.

### Parameterizing an age-structured model for cadaver-turnover in graveyards

From fieldwork in Thailand on a similar system of *O. unilateralis s.l.* infecting *Camponotus leonardi* [7] and from observations of newly infected ants in Brazil we estimated the duration of the different parasite life stages. The first freshly killed stage, from death to the first signs of a stroma, takes *ca.* 4 days, for parasites in the stromal stage we arrived at a wide range of stage-duration times (7–30 days), and we inferred that parasites with sexually mature fruiting bodies were at least 20 days old. These ranges are likely to vary across the season, as growth conditions for fungi probably are proportional to rainfall. To remain conservative when parameterizing our model (see below), we used a relatively crude timescale assuming that development from host death to stroma appearance takes one week, and development from stroma to sexual maturity takes four weeks.

Adopting the approximate approach described above has the advantage that estimates apply to both *Ophiocordyceps unilateralis* infections in Thailand and *O. camponoti-rufipedis* infections in Brazil, allowing us to supplement missing data in our present study. Data from Thailand obtained by following 17 individuals for 10 months with intervals of two months produced approximate figures for the risk of *O. unilateralis s.l.* becoming hyperparasitized at a given age. We found that 30/31 *O. unilateralis s.l* monitored for 18 months became infected by hyperparasitic fungi. Based on our field observation we estimated that mature parasites persist for at least 4 weeks before being hyperparasitized (DPH, unpublished data). Finally, we assumed that the accumulation of freshly dead cadavers was proportional to the number of spore-producing parasites with mature fruiting bodies, as our measurements would show that neither immature stromata nor mature but hyperparasitized fruiting bodies produced any spores.

**Figure 1:**
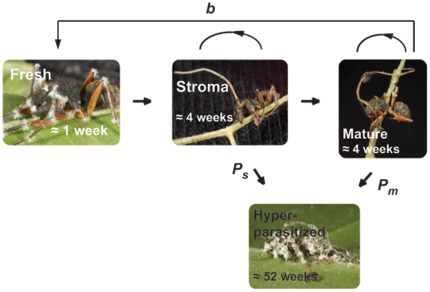
Idealized parasite life-cycle. Boxes indicate life stages (fresh, stroma, mature and hyperparasitized) and arrows indicate transitions between stages. New cadavers enter the system with birth rate *b* and remain in the ‘fresh’ stage for a week on average. They then move to the ‘stroma’ life stage and stay there for an average of four weeks, during which a proportion is lost to the ‘hyperparasitized’ life stage at rate *P_s_*. Those individuals that move to the mature stage spend on average four weeks there, during which a proportion is lost to the ‘hyperparasitized’ life stage at rate *P_m_*. Individuals in the hyperparasitized stage remain on here for an average of 52 weeks before being lost.

We formalized the “life-cycle” of parasitized cadavers as illustrated in Figure 1, where arrows represent transitions between life stages. We assumed that the rate of new infections, *b*, is determined by the availability of live ants and is constant over time (assuming no colony growth or decline). We further assumed that cadavers in the stromal stage would be hyperparasitized at a rate *P_s_* and cadavers in the mature stage would be hyperparasitized at rate *P_m_*; these would then abort the completion of these stages and transfer them to the effectively sterile hyperparasitized stage (see data below). Writing the number of cadavers in each class as a vector N = (Fresh, Stroma, Mature, Hyperparasitized)', we then summarized the cadaver “life cycle” as a population transition matrix given by equation 1:
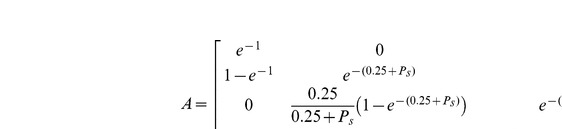
In this matrix (Equation 1) time steps proceed in multiples of one week and development rates are such that the mean time in each class is one week for the freshly killed cadaver class, and four weeks for both the stromal and the sexually mature fruiting body stage.

If we assume that the rates of transition between classes in *A* are constant through time then the long-term stable stage distribution of the matrix *A* gives the expected distribution of cadavers observed in each class. To estimate the unknown parameters of *A* (*b, P_s_,* and *P_m_*), we found the values that minimized the sum of squared differences between the expected stable stage distribution of *A* and the stage distribution of cadavers observed in the field. This allowed us to subsequently estimate the graveyard growth rate, *λ*, accounting for the unhyperparasitized part of the graveyard, as the dominant eigenvalue of the transition matrix [33], [34].

### Adding variation in overall growth rates

The growth rate of graveyards (*λ*) and the developmental stage distribution in graveyards depends on the assumed fungal development rate, which in turn depends on temperature and humidity [35], [36], [37]. We first explored the sensitivity of the estimated parameters *b*, *P_s_*, *P_m_* and *λ* to effects of variation in the assumed fungal developmental rate. This was done by varying *Ophiocordyceps* development rates relative to the fitted model by ±50% from the original fitted value, by incorporating the parameter *θ* (ranging from 0.5 to 1.5, rather than being fixed at 1) to Equation 1, under the assumption that all parasite life stages would be affected equally. We then set up four alternative versions of Equation 1 to further explore the effects of seasonal variation in the fungal developmental rate on the graveyard growth rate and the proportion of the graveyard that has escaped hyperparasitism in relation to faster (*θ*>1) or slower (*θ*<1) overall fungal development. We hypothesized that seasonal variation could affect fungal life history in three different ways by: 1. only affecting parasite developmental rate, 2. affecting parasite and hyperparasite developmental rates similarly so they become positively correlated and 3. affecting the inflow rate of new cadavers *b* by a positive correlation between time spent in the mature life stage and new ants infected. The four alternative scenarios capture the different combinations of these potential effects of variation in fungal development rate. In scenario 1A and 1B the inflow rate of new cadavers is assumed to be uncorrelated with the parasite development rate, while cadaver inflow rate is assumed to be positively correlated with parasite development rate in scenario 2A and 2B. In scenario 1A and 2A hyperparasite development is uncorrelated with parasite development time, while the variables are positively correlated in scenario 1B and 2B (Table 1).

**Table 1:** Four alternative scenarios of the impact of variation in fungal development rate for *Ophiocordyceps* and hyperparasitic fungi.

Model	*Ophiocordyceps* development rate (all stages)	Infection rate of new cadavers (*b*)	Hyperparasite development rate	Equations
Scenario 1A	Correlated with the environment	No effect	No effect	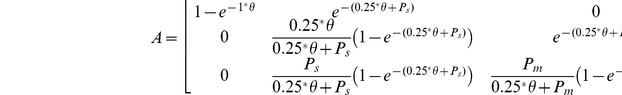
Scenario 1B	Correlated with the environment	No effect	Correlated with the environment	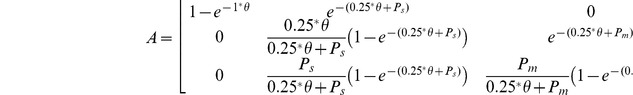
Scenario 2A	Correlated with the environment	Correlated with the environment	No effect	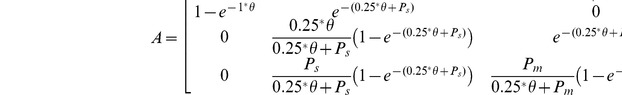
Scenario 2B	Correlated with the environment	Correlated with the environment	Correlated with the environment	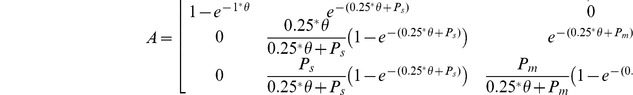

In scenario 1A and B the infection rate (*b*) is uncorrelated with parasite developmental rate while the rates are correlated in scenario 2A and B, meaning that the time the parasite spends in the mature life stage, as determined by development rate, is positively correlated with the rate at which new infected individuals appear. In scenario 1A and 2A the hyperparasite developmental rate is uncorrelated with parasite development rate while the rates are correlated in scenario 1B and 2B, implying that environmental factors determining fungal growth affect the parasite and hyperparasites in the same way.

## Results

### Life stage distribution of dead infected ants in graveyards

**Figure 2:**
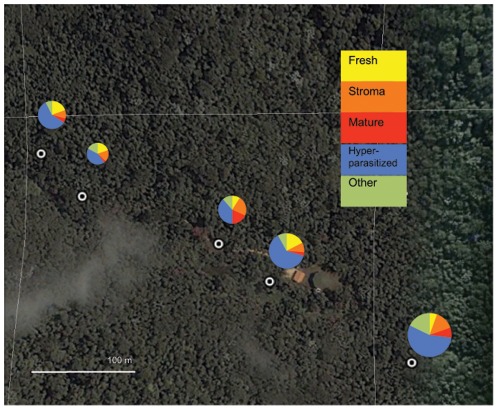
Graveyard location and cadaver distribution. Aerial photo (from GoogleEarth) of the sampling area in Mata do Paraíso with the five graveyards marked and the distribution of parasite life stages plotted as pie charts. A total of 432 dead infected ants were encountered, distributed with 41, 35, 44, 149 & 163 individuals, respectively, in graveyard 1–5.

In the five graveyards we found a total of 432 dead infected ants; 12.5% were fresh, 12.9% carried a stroma, 6.5% were mature, 55.4% were hyperparasitized, and the remaining 12.7% were damaged with no obvious fungal growth (Fig. 2).

None of the mature parasite fruiting bodies (0/15) dispersed spores at ambient temperature and humidity at the time of our spore collection in the forest. However, after exposure to high humidity simulating nights of heavy rainfall in the forest, 42% (13/31) of the mature parasite fruiting bodies were shooting sexual spores (ascospores) in the lab.

### Model fitting

**Figure 3:**
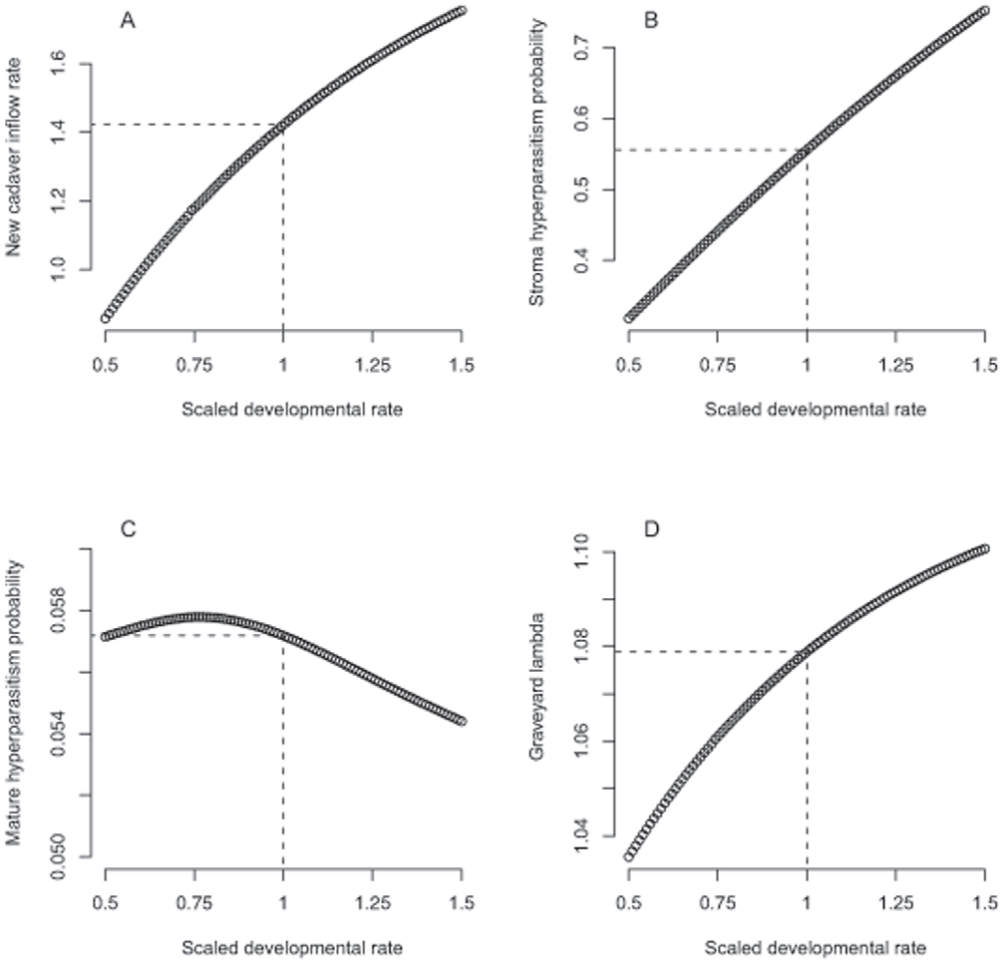
Sensitivity analysis of the estimated life-history parameters. The analysis was based on the stage structured graveyard growth model that fitted the empirical data best. Panel A: the new cadaver inflow rate *b*, panel B: the hyperparasitism rate in the stromal life state *P_s_*, panel C: the hyperparasitism rate in the mature life stage *P_m_*, Panel D: the graveyard growth rate *λ*. The variation in fungal developmental rate from 50% to 150% is plotted along the x-axes, relative to the average fungal development rate that was estimated from the field data (here represented by the relative value of 1). Dashed lines connect the expected mean for the y-axis estimate with this overall mean development rate.

We fitted the stage-structured graveyard growth model to the observed distributions of parasite life stages and performed simulations assuming that fungal developmental rates ranged from 50% to 150% of our estimated means to evaluate the sensitivity of parameter estimates to the assumed developmental rates. The estimated cadaver inflow rate *b* was 1.42 new cadavers per mature cadaver, varying from 0.85 to 1.75 across the total range of 0.5 to 1.5 times the mean developmental rate (Fig. 3A). The estimated probability of hyperparasitism among parasites in the stromal stage *P_s_* was 0.55, varying from 0.31 to 0.75 across the total range of 0.5 to 1.5 times the mean developmental rate (Fig. 3B). The estimated probability of hyperparasitism among parasites in the mature stage *P_m_* was 0.057, and relatively invariant across the range of developmental rates. This suggests that the probability of new hyperparasitism of mature parasites is very limited for the four weeks that the parasite is assumed to spend in this life stage (Fig. 3C). The graveyard growth rate *λ* for the model showing the best overall fit with all parameter values was 1.07 and had a very small range of variation (1.035–1.100) across the range of 0.5 to 1.5 times the mean developmental rate, indicating that the observed stage distribution was consistent with a relatively slow graveyard growth rate (Fig. 3D).

### Growth and longevity trade-off

**Figure 4:**
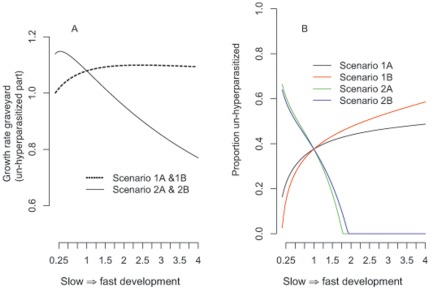
Modelling of graveyard growth rate and degree of hyperparasitism. Graveyard growth rate *λ* (panel A) and proportion of cadavers non-hyperparasitized (panel B) as a function of developmental rate (x-axis) for the four different modelled scenarios. The variation in fungal developmental rate from .25% to 400% is plotted along the x-axes, relative to the average fungal development rate that was estimated from the field data (here represented by the relative value of 1). Panel A: The graveyard growth rate, which only accounts for the non-hyperparasitized individuals, is identical in scenario 1A and 1B, and is >1 across all developmental rates but peaks at fast to intermediate developmental rate. Scenario 2A and B are also identical, with negative growth rates at fast development rates but peaks with growth rates >1 at intermediate to slow developmental rates. Panel B: The four scenarios differ in the proportion of non-hyperparasitized cadavers across the developmental range. Scenario 1A and 1B have high rates of hyperparasitism at slow developmental rates. Note that as development rates increase, a greater proportion of cadavers escape hyper-parasitism in scenario 1B due to the faster senescence of the hyperparasitized cadavers. Scenario 2A and 2B show have increasing rates of hyperparasitism as the developmental rate increases due to the relative decrease in the inflow of new cadavers.

Exploring the four different scenarios for implementation of variation in overall fungal development rate (the x-axis in Fig. 4) showed that, as expected, slow development increases the time spent in the mature, infectious stage and the likelihood of a susceptible ant coming in contact with spores, while fast development increases the likelihood of reaching the mature stage prior to hyperparasitism. In scenario 1A and 1B, where the inflow rate of new cadavers is uncorrelated with the *Ophiocordyceps* developmental rate, the overall growth rate of the graveyard remains >1 for the total range of fungal development rates (dashed curve, Fig. 4A; scenario 1A and 1B give the same result, as do 2A and 2B, because they only differ in whether the probability of hyperparasitized individuals remaining in the graveyard is correlated to the parasite development rate or not, which in the model does not affect the graveyard growth rate). However, if faster fungal development also results in lower infectivity due to reduced likelihood of ant encounters (scenario 2A and 2B), then the graveyard growth peaks at even slower rates of fungal development and rapidly declines when developmental rates increase (solid curve, Fig. 4A).

The four scenarios differ in the proportion of cadavers that remain free of hyperparasites (Fig. 4B). If parasite and hyperparasite developmental rates affect only the transitions among cadaver categories (1A and B; black and red curves), then faster development rates result in fewer hyperparasitized cadavers (Fig. 4B). Faster developmental rates in the hyperparasite lead to faster senescence of hyperparasitized cadavers and thus a greater proportion of non-hyperparasitized cadavers (i.e. the difference between scenario 1A and 1B, Fig. 4B). If the cadaver inflow rate is positively correlated with the time spent in the mature stage (scenario 2A and 2B, green and blue curves) the proportion of non-hyperparasitized cadavers is maximized at slow developmental rates as fast development leads to relatively low replenishment of fresh, non-hyperparasitized cadavers.

## Discussion

### Low density and limited interaction efficiency between infective parasites and susceptible hosts

We found that only *ca*. 6.5% of the *O. camponoti-rufipedis* fruiting bodies were effectively producing spores, as most dead ants were sterile because they were immature (25.5%), damaged (12.7%) or hyperparasitized (55.4%) by other fungi that are not pathogens of ants. Field and lab trials further indicated that only 42% (13 out of 31 tested) of the apparent fertile fruiting bodies were shooting spores at a particular time interval, illustrating that detailed micro-environmental conditions also matter. Upon dissection some apparently ‘healthy’ *O. camponoti-rufipedis* cadavers were found to be invaded by larvae of small unidentified arthropods (SBA and DPH, unpublished data). This may also have reduced the probability of the parasite reaching maturity and would have moved a number of them to the hyperparasitized category. This demonstrates that most cadavers are not infectious to foraging ants and implies that disease pressure at the colony-level is much lower than the high numbers of dead graveyard ants suggest.

In addition to the low number of infective parasites, only a small percentage of the ant colony members are actually available as targets for *Ophiocordyceps* spores, as all brood and most workers remain inside the safe nest boundaries, so that only foragers face the risk of encountering spores [18], [19], [20]. Transmission between ants within a colony seems unlikely due to the large spore size (100–200 µm in length compared to e.g. the 2–9 µm of *Beauveria* and *Metarhizium* spores [38]). The local interaction-interface between parasite and host is therefore likely to be limited, so colony-level infections can only be stable when graveyards continue to grow until a steady state that maintains host and parasite individuals at relatively constant densities of chronic reinfection of ants from the same colony. The finding of a graveyard growth rate just above one by the modelling approach supports such a scenario.

### The logic of iteroparous reproduction in *Ophiocordyceps*

As outlined in the Introduction, iteroparous reproduction tends to be evolutionary stable when externally imposed juvenile mortality is high relative to adult mortality [30]. Juvenile mortality of the parasite may occur at three different stages: the produced spores, the infected ants when they are still alive, and the immature parasites developing in ant cadavers. We do not have an estimate of the proportion of infected ants not ending up as graveyard cadavers, but we note that these individuals unaccounted for will add both to the mortality of parasites and the parasite pressure on the colony (by ant death elsewhere or cost of clearance). However, the UV sensitive, hyaline thin-walled, and thus desiccation prone, ascospores of *O. camponoti-rufipedis* [24] seem to fit this characteristic. These spores might thus reflect an adaptive life-history strategy of producing short-lived spores released asynchronously over a long period rather than long-lived spores in synchronous flushes for a short time. While the majority of entomopathogenic fungal spores are somewhat fragile, other species have different strategies. Some generalists such as the well-studied genera *Metarhizium* and *Beauveria* that are anamorphs of *Cordyceps*-like teleomorphs, combine rapid semelparous asexual reproduction with the production of persistent conidiospores. This is also the case for the ant manipulating fungus *Pandora sp*., infecting *Formica* hosts (J. Malagocka & A.B. Jensen, unpublished). The model is build on the assumption that ant infecting *Ophiocordyceps* do not produce persistent resting spores and do not grow naturally in the absence of ant hosts, which all available evidence supports. However, it is worth noting that *Metarhizium* and *Beauveria* are also increasingly suspected to have ‘hidden lives’ as endophytes of leaves and in the rhizosphere, with the possibility to produce spores outside the bodies of insect hosts [39], [40] and probably also with well-adapted saprophytic phases surviving on chitinous debris in the soil [41].

Our model suggests that also the immature fruiting body stages are highly vulnerable, but here the external factors are biotic rather than abiotic, because hyperparasitism risk is high in the stromal parasite life stage (*ca*. 55%; Fig. 3B). In addition, our model confirmed that mortality in the reproductive stage was low, with hyperparasitism being negligible in the mature life-stage (*ca*. 5.5%; Fig. 3C). This is under the assumption that the mature life stage has a duration period of one month: in the field in Thailand mature fruiting bodies were observed to accumulate hyperparasites after this time period but we speculate that spore production may have ceased at this stage. A low risk of hyperparasitism may well be related to mature fruiting bodies expressing a much more efficient immune defence than the rapidly growing stromata, because it takes time for the growing parasite mycelium to compartmentalize the dead host body into specific fungal tissues with complementary roles in protecting the elaborate fruiting body structures that produce the spores [7]. Fungal immune defences are poorly understood (but see eg. [42]), but *Ophiocordyceps* fungi are known to produce a range of secondary metabolites that may be relevant for maintaining cadavers [43]. Thus, the likelihood for spores to survive, reach their target and infect – resulting in the production of stromata - is very low, but a fruiting body that made it towards maturity is worth maintaining for a long time and likely to be required for securing parasite transmission.

Though not known in any detail, morphological structures such as stalks and spore producing bodies in a range of the other hypocrealean fungi, such as those infecting lepidoteran and coleopteran larvae and spiders, indicate that iteroparity occurs more widely [5]. While our knowledge of these groups is cursory the life-histories of these parasites also appear to be characterized by a high degree of host specificity and limited contact between hosts and infective spores. All *Ophiocordyceps* appear to have evolved and be most diverse in tropical regions, with the most pronounced centre of diversity in tropical South East Asia [5]. This suggests that the evolution and maintenance of iteroparity in these obligate insect pathogens is primarily related to host characteristics and parasite specificity, and that ant hosts have merely required the additional evolution of the well-known extended phenotypes where infected workers are manipulated to leave their nests.

Our model also explored whether and how it matters that the time spent in the mature parasite stage is positively correlated with cadaver inflow rate (scenario 2A and 2B) or not (scenario 1A and 1B). Such correlations are likely to exist because the number of new infected ants should be some continuously increasing function of the number of spores produced in graveyards, which in turn should be positively correlated with the reproductive life span of mature fruiting bodies. As lifespan would likely trade-off against development rate [44], this suite of traits would then also be accompanied by slow development. Our model confirms that positive correlations between fruiting body life span and cadaver inflow rate maximize graveyard growth and the proportion of cadavers that escape being hyperparasitized, provided development is slow (Fig. 4). However, when fruiting body life span and cadaver inflow rate are not correlated, faster rates of development appear to be optimal for parasite reproduction (scenario 1A and 1B). We believe that correlations between parasite and hyperparasite developmental rates are likely to occur as fluctuations in temperature and humidity may affect different fungal species similarly. However, such correlations may also vary considerably because hyperparasite growth is generally faster than *Ophiocordyceps* growth, due to their simplified morphology.

### Graveyard growth and disease spread across spatial scales

Our estimates of graveyard growth rates just above 1 are consistent with colony-specific aggregations of dead ants being sustainable, with each mature parasite on average producing slightly more than one new mature parasite. This result is important because it illustrates that population-level disease dynamics of parasites such as *Ophiocordyceps* can only be understood when considering the colony rather than the individual ant as the host [11], [12]. If ant workers die close to their nest and end up infecting their younger siblings, this is equivalent to intracolony or within-host transmission [14], [15], [17]. By contrast, true horizontal transmission would then be restricted to spores produced by parasites of one colony infecting workers of another colony. This could either be achieved by rare infected workers dying much further away from their colony than the ‘resident’ graveyard, or by spores produced in graveyards occasionally dispersing over much longer distances.

Long distance spore dispersal seems unlikely as the sexual spores are exceptionally large (100–200 µm in length) and, therefore, probably not easily dispersed by wind. Nevertheless, we now know that *O. unilateralis s.l.* also has a range of asexual stages (synanamorphs) with spores seemingly designed for either persistence or aerial dispersal; although *O. camponoti-rufipedis* is the exception and only produces a single anamorph [24], [26]. Why related *Ophiocordyceps*-*Camponotus* associations in the same habitats produce a variety of synanamorphs remains unexplained [24]. Interestingly, in the ‘true *Cordyceps*’, the long, thin, multiseptate ascospores break up into many part-spores (averaging *ca*. 5 µm in length), as they are forcibly released from the stroma, which could be an adaptation to long-distance dispersal. However, if horizontal transmission would primarily depend on the movement of the infected ants themselves, this would suggest the intriguing possibility of disruptive selection on parasite extended phenotypes for both short and (occasionally) long-distance dispersal. Infected ants should then either die very close or relatively far from their colony because ant territories are geographical mosaics with most if not all interactions being restricted to nearest neighbour colonies [45], [46], [47]. Such distances between ‘host patches’ of group-living species in comparison to solitary hosts has been suggested to decrease overall disease susceptibility in spite of locally high host densities [48]. Future studies may address this by assessing the gene flow within and between graveyards by looking at the genetic diversity of the dead hosts and their parasites.
